# Staging of Laryngeal and Hypopharyngeal Cancer: Computed Tomography versus Histopathology

**Published:** 2018-07

**Authors:** Bhagyashree Jaipuria, Deviprasad Dosemane, Panduranga M Kamath, Suja S Sreedharan, Vijendra S Shenoy

**Affiliations:** 1 *Department of Otorhinolaryngology, Kasturba Medical College, Mangalore, MAHE. India.*

**Keywords:** Computed Tomography, Hypopharyngeal cancer, Histopathology, Laryngeal cancer

## Abstract

**Introduction:**

Computed tomography (CT) imaging is the choice of investigation for evaluation of extent of tumor under the mucosa, locally and regionally. This study was undertaken to assess the accuracy of preoperative CT imaging in the staging of carcinoma of the larynx and hypopharynx.

**Materials and Methods:**

In this retrospective study, all cases who were clinically (c) staged T3–T4 and who underwent surgery were evaluated. The sensitivity (Sn), specificity (Sp), positive predictive value (PPV), negative predictive value (NPV), and accuracy (Ac) of a preoperative CT scan to detect cartilaginous penetration and spread outside the larynx was evaluated. Clinical T-stage by CT imaging was compared with postoperative histopathological T-stage.

**Results:**

There were 22 (96%) male patients and one (4%) female patient. Patients were aged from 48 to 64 years (mean, 56 years). Twenty (87%) patients had laryngeal carcinoma, whereas three (13%) had hypopharyngeal carcinoma. Four (17%) patients had T3 tumors and 19 (83%) had T4a lesions. Preoperative imaging over-staged one of five pathological (p) T3 cases and three out of three pT2 cases, and was accurate in the remaining 19 cases. In a comparison of the results of imaging with histopathological T-classification, T-staging was found to be 82.6% accurate.

**Conclusion:**

CT is a precise and non-invasive imaging method with high sensitivity but relatively lower specificity because of increased false positives. CT imaging may be useful to guide the choice of surgical procedure, which can heavily impact a patients’ quality of life.

## Introduction

Cancer of the larynx is so common that it accounts for one in every five cases of malignancy of the head and neck area, with squamous cell carcinoma (SCC) accounting for the majority (1,2). Up to 40% of patients visit doctors at a late stage of the disease (3). In total, 65–85% of cancers of the hypopharynx involve the pyriform fossae, 10–20% arise from the posterior pharyngeal wall, and 5–15% from the postcricoid region (4).

The location and extent of laryngeal and hypopharyngeal malignancies are mainly evaluated by endoscopy. A combination of video laryngoscopic pictures with radiological imaging to assess the extent of the tumor under the mucosa, locally and regionally, improves the accuracy of the staging of the tumor and influences the management plan in these patients (5). Cancer of the larynx and hypopharynx, particularly T4a lesions involving the cartilage and spreading out of the larynx, needs accurate evaluation, because management can have a huge influence on the quality of life of the patient. Therefore, reliable imaging tools are necessary. Computed tomography (CT) is the preferred imaging modality as it provides good spatial and temporal resolution. Cartilage destruction can be better identified through findings such as erosion, lysis, and transmural spread out of the larynx, but tumors that take up iodine are often difficult to differentiate from non-ossified cartilage.Hence the present study was performed at our tertiary care center to assess the accuracy of preoperative CT in laryngeal and hypopharyngeal carcinoma staging by comparison with histological cross-sections of postoperative specimens.

## Materials and Methods

Data from patients with SCC of the larynx or hypopharynx, who underwent surgery between September 2012 and August 2017, were studied. The eligibility principal was to include histopathologically diagnosed with laryngeal or hypopharyngeal SCC and clinical (c) T3–T4 tumors. Patients who received neoadjuvant chemotherapy or radiotherapy were excluded. 

A contrast-enhanced CT (CECT) scan was performed (within 2–4 weeks before the surgical procedure) for all patients from the skull base to the mediastinum. The field of view (FOV) was 16 to 20 cm, as per the length of the patient’s neck. All CECT films were read by one radiologist only who was unaware of the endoscopic findings and other pathologic data.

A single pathology staff member sectioned the tumors into axial, serial slices parallel to the vocal cords at 2-mm intervals, and then studied the slides. Histopathological reporting was performed according to the latest College of American Pathologists (CAP) protocols, with larynx cancer as per CAP protocol larynx version 3.0.0. and hypopharynx cancer as per CAP protocol pharynx version 3.3.0.0. The pathologist was blinded to the endoscopic findings and radiologic data. Quantitative data were described in the form of mean and standard deviations. Study groups were compared using the paired t-test as per the results of the normality test. Qualitative data were described using frequency and percentage. The Chi-square test was used to look for the association between the study groups. A p-value less than 0.05 was considered significant**. **MS Excel and SPSS ver. 20 were used for statistical analyses.

## Results

The demographic, clinical, and pathological characteristics of the cases are shown in Table 1. 

**Table 1:** Patient characteristics (n=23

**Variable**	
Median age, years (range)	56 (48–64)
Gender	
Male/female, n	22/1
Site of primary tumor	
Larynx, n Supraglottis/glottis/subglottis/transglottic Hypopharynx	20 5/12/0/3 3
Pathologic differentiation, n Well/moderately/poorly/not specified	9/13/0/1
cTNM classification (AJCC 6th ed.), n T3/T4 a	4/19
Pathologic T-staging, n pT2/pT3/pT4a	3/5/15

**Fig 1:**
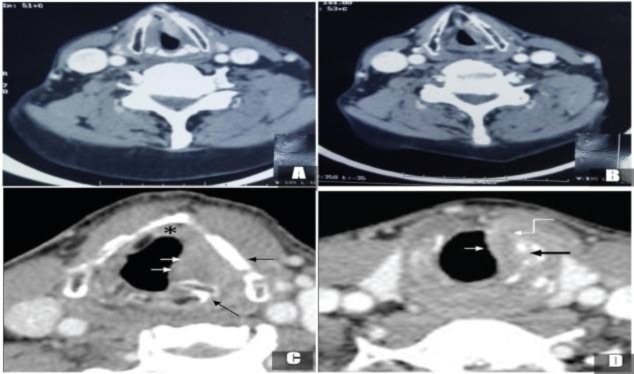
Axial sections of contrast-enhanced CT images of glottic SCC.

Thyroid cartilage destruction was reported in 23 cases by imaging and histopathology, while CT imaging identified 13 (76.5%) of 17 cases of histopathologically confirmed thyroid cartilage destruction (Fig.1). 

A. Glottic mass in the left vocal cord involving anterior two thirds, extending up to the anterior commissure. Thyroid and cricoid cartilages appear normal.

B. Glottic mass in the left vocal cord involving anterior two thirds extending up to the anterior commissure and invading left paraglottic fat (arrow).

C Advanced glottic Ca. Tumor mass (thin white arrows) with disease in the posterior commissure (oblique black arrow), reaching up to anterior commissure (asterisk) and cricoid erosion (horizontal black arrow).

D Advanced glottic SCC. Tumor mass extending into the subglottis (thin white arrow) with irregularity of cricothyroid membrane, ELS (white elbow arrow), and erosion of cricoid cartilage (black arrow).

Considering histopathology as the “gold standard”, the accuracy (Acc), positive predictive value (PPV), and negative predictive value (NPV) of imaging for detection of penetration of the thyroid cartilage (n=23) were 73.9%, 76.5%, and 66.7%, respectively, as shown in Table 2.

**Fig 2:**
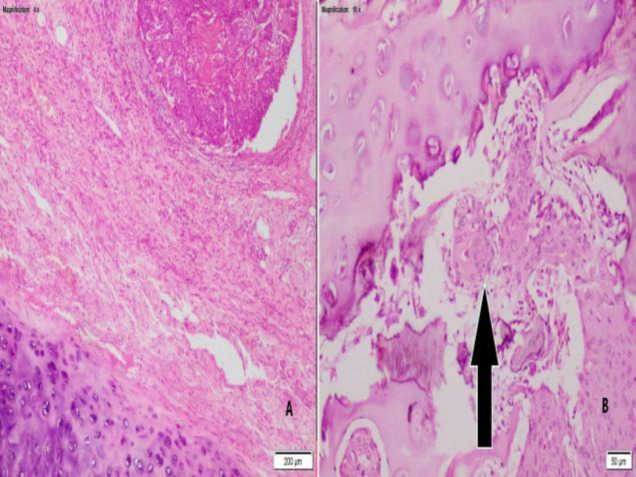
A. Hematoxylin and eosin-stained section from the tumor tissue shows cartilage to be free from infiltration (4×). B. Hematoxylin and eosin staining of SCC infiltrating cartilage (black arrow) of the patient with glottic cancer (10×).

Involvement of the arytenoid and cricoid was pathologically reported among 12 and 13 cases, respectively (Fig.2); this review was not performed if no sample was taken from the cricoid or arytenoid. The sensitivity (Sn), specificity (Sp), Acc, PPV, and NPV of the scans in determining the destruction of these two types of cartilage are shown in (Table 2). 

**Table 2:** Comparison of preoperative CT and postoperative histopathological findings in patients

	TP	FP	FN	TN	Sn (%)	Sp (%)	PPV	NPV	Acc
**Cartilage destruction**									
Thyroid cartilage penetration (n=15)	13	4	2	4	86.7	50	76.5	66.7	65.2
Cricoid cartilage destruction (n=13)	3	2	1	10	75	83.3	60	90.9	56.5
Arytenoid cartilage destruction (n=12)	4	3	1	8	80	72.7	57.1	88.9	52.2
Extra-laryngeal spread (n=9)	2	4	2	7	62.5	92.9	83.3	81.3	78.3
Subglottic extension (n=18)	5	1	3	13	50	63.6	33.3	77.8	39.1
**Compartmental involvement**									
Paraglottic space (n=19)	12	2	2	7	85.7	77.8	85.7	77.8	82.6
Pre-epiglottic space (n=10)	3	4	2	7	60	63.6	42.9	77.8	43.5

TP=true-positive, FP=false-positive, FN=false-negative, TN=true-negative, PPV=positive predictive value, NPV=negative predictive value, Sn=Sensitivity, Sp=Specificity, Acc=Accuracy

**Fig 3:**
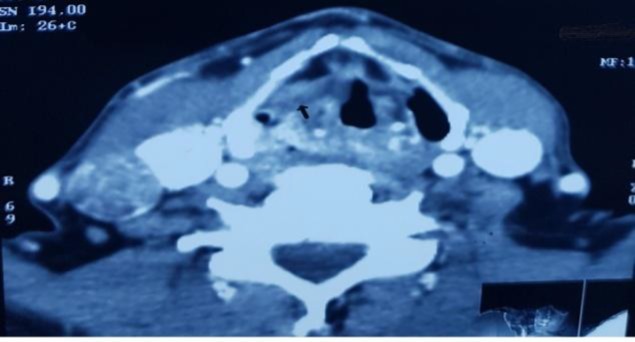
Axial section of CT scans showing right pyriform sinus SCC with extension into the paraglottic fat anteriorly (arrow).

**Fig 4:**
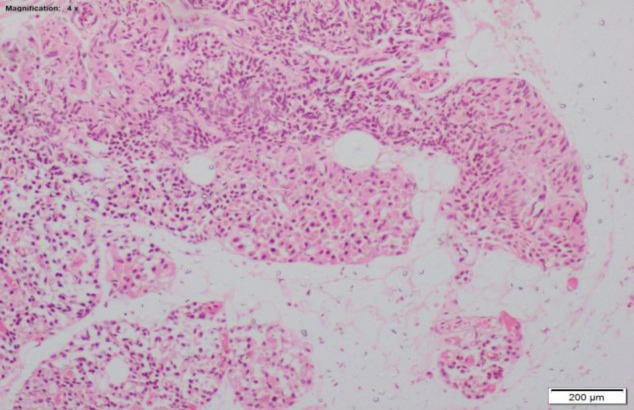
Hematoxylin and eosin-stained section showing mucosa, submucosal glands, and cartilage with no evidence of tumor. Pre-epiglottic pad of fat is seen (4×).

Paraglottic space (PGS) and pre-epiglottic space (PES) involvement was shown among 14 and seven patients, respectively, by imaging (Fig.3) and among 14 and five cases, respectively, by histopathology (Fig.4). Subglottis spread of the lesion was shown in six cases on CT imaging and eight cases on histopathology. The Sn, Sp, Acc, PPV, and NPV of imaging in determining the involvement of PGS and PES are summarized in Table 2. Extra-laryngeal spread (ELS) was seen in six patients on imaging and in four patients on histopathology. Two (73%) of four cases with histopathologically confirmed ELS were identified by CT imaging. The Acc, PPV, and NPV for detection of ELS by imaging was 81.8% (95% confidence interval, 65–91%), 83.3% (41–97%), and 81.3% (63–91%), respectively (Table.2).

Clinical T-stage by CT imaging was compared with postoperative histopathologic T-stage. Preoperative imaging over-staged one of five pT3 lesions and all of three pT2 lesions, and was accurate in the remaining 19 patients (Table.3). If the imaging results are compared with histopathology, T-staging was 82.6% accurate.

**Table 3:** Association of clinical and pathologic staging in patients

Clinical Staging	N	Pathologic Staging			
		pT1	pT2	pT3	pT4a
**T1**	0	-	-	-	**-**
**T2**	0	-	3	-	**-**
**T3**	4	-	-	5	**0**
**T4a**	19	-	-	-	**15**

## Discussion

The appropriate T-stage of a laryngeal carcinoma can be obtained by obtaining accurate information relating to extension into the sub-anatomical areas and cartilaginous involvement by the primary tumor. Many studies have stressed that CT scans are important in the pretreatment staging of carcinoma of the larynx, but only a few have studied the role of CT in assessment of the sub-anatomical areas of the larynx (6–12).

The radiological appearance of the cartilages of the larynx may vary according to the amount of ossification or the volume of marrow fat in the medulla (13). Bertrand et al. concluded that a CT scan is the first-line radiological evaluation for early glottic carcinomas. Later, they used magnetic resonance imaging (MRI) to observe involvement of doubtful regions such as the subglottis, anterior commissure, and arytenoids (14).

Atlanoğlu et al. performed a study to see how accurate multi-slice CT is in comparison with T-stage as determined by histopathology of a carcinoma of the larynx (15). His study included 27 men and a single woman with a mean age of 59.57 years (range, 36–77 years). Our study also had a male preponderance of 95.7%, with a mean age of 56.09 ± 8.02 years. In the same study, the site of the primary tumor in the majority of patients was supraglottic, followed by glottic, glotto-subglottic and transglottic (4-6,13). The site of the primary tumor in the majority of patients in our study was glottic (52.2%), followed by supraglottic (21.6%), transglottic (13.1%), and hypopharyngeal (13.1%).

Beitler et al. studied the accuracy of pretreatment CT imaging in determining the need for laryngectomy initially for advanced carcinoma of the larynx (16). He reported that CT imaging showed thyroid cartilage involvement in 23 patients and ELS in 27 patients, whereas histopathology showed 12 specimens with cancer invading and 29 specimens of cancer penetrating the thyroid cartilage and 45 specimens with ELS.

 Seventeen (59%) of 29 patients with histopathology confirmed thyroid cartilage penetration and 22 (49%) of 45 patients with histopathology confirmed ELS were identified by CT imaging. Histopathologically proven ELS with no thyroid cartilage involvement was seen in eighteen (40%) of 45 patients.

The role of CT imaging in detecting involvement of the cartilages in early glottic tumors was evaluated by Hartl et al. (17). This study reported an Sn of 10.5% and Sp of 94%, and overestimated the invasion of cartilages in patients with lesions involving the commissure and in lesions with impairment of cord movement. The authors stressed that CT imaging was unable to evaluate focally invaded inner perichondrial lesions in the thyroid cartilage.

In a study conducted by Ryu et al. (18), 23 (72%) of 32 patients with histopathologically confirmed thyroid cartilage destruction and 24 (73%) of 33 patients with histopathologically confirmed ELS were identified by imaging. The PPV for thyroid cartilage destruction and ELS were 70% and 80%, respectively. Pretherapeutic CT over-staged six of 46 pT3 lesions and all eight pT2 lesions, while it under-staged seven of 40 pT4 lesions. The clinical T-staging by CT was 78% accurate. In our study, statistical values for pretreatment CT imaging were as follows: for thyroid cartilage penetration (n=23) Sn 86.7%, Sp 50%, PPV 76.5%, NPV 66.7%, and Acc 73.9%; ELS (n=22) Sn 62.5%, Sp 92.9%, PPV 83.3%, NPV 81.3%, and Acc 81.8%. In our study, preoperative CT was most accurate in the detection of the paraglottic space (82.6%) and least accurate in the detection of the subglottic space (60%), as compared with postoperative histopathological findings. According to Agada et al. (19), on comparison of radiologic staging with histopathologic specimens, 45% cases got up-staged and 10% got down-staged on CT scan.

In a paper by Atlanoğlu et al., the staging was as follows: two cases (7.1%) of T1a, three (10.7%) of T2, 14 (50%) of T3, and nine cases (32.2%) of T4a (15). In comparison with histopathologic staging, clinical staging was accurate in four (14%) patients. Twenty-four patients were under-staged by clinical examination. The matching between clinical staging and pathological staging was fair (κw=0.06). Twenty-three (82.1%) patients were accurately staged. The match between T-stage on imaging and histopathology was good (κw=0.773). In four (14.2%) (2T4a,2T3) cases, there was an overestimation of CT and in one (3%) case, there was underestimation.

In a study conducted by Allegra (20) et al, 14 of 20 patients (70%) were accurately staged and six patients were under-staged. Two lesions which were cT1a on CT were pT1b, two lesions which were cT1a were pT3, and two lesions were missed on CT. CT was found to have only 40% Sn but 100% Sp for lesions of the anterior commissure, cartilage of the larynx and PGS. In our study, it was observed that clinical staging was accurate in 19 of 23 patients, with an overall accuracy of 82.6%. Three T2 stage and one T3 stage cases were over-staged to T4.

## Conclusion

CT is a precise and non-invasive imaging method for the staging of laryngeal and hypopharyngeal carcinoma with high sensitivity. Although CT has relatively less specificity because of increased false positives, it may be useful to dictate choice of surgical procedure by identifying clinically significant thyroid cartilage penetration.
